# Generation and analysis of expressed sequence tags from a cDNA library of the fruiting body of *Ganoderma lucidum*

**DOI:** 10.1186/1749-8546-5-9

**Published:** 2010-03-16

**Authors:** Hongmei Luo, Chao Sun, Jingyuan Song, Jin Lan, Ying Li, Xiwen Li, Shilin Chen

**Affiliations:** 1 Institute of Medicinal Plant Development, Chinese Academy of Medical Sciences and Peking Union Medical College, 151 Malianwa North Road, Haidian District, Beijing 100193, PR China

## Abstract

**Background:**

Little genomic or trancriptomic information on *Ganoderma lucidum *(*Lingzhi*) is known. This study aims to discover the transcripts involved in secondary metabolite biosynthesis and developmental regulation of *G. lucidum *using an expressed sequence tag (EST) library.

**Methods:**

A cDNA library was constructed from the *G*. *lucidum *fruiting body. Its high-quality ESTs were assembled into unique sequences with contigs and singletons. The unique sequences were annotated according to sequence similarities to genes or proteins available in public databases. The detection of simple sequence repeats (SSRs) was preformed by online analysis.

**Results:**

A total of 1,023 clones were randomly selected from the *G*. *lucidum *library and sequenced, yielding 879 high-quality ESTs. These ESTs showed similarities to a diverse range of genes. The sequences encoding squalene epoxidase (SE) and farnesyl-diphosphate synthase (FPS) were identified in this EST collection. Several candidate genes, such as *hydrophobin*, *MOB2*, *profilin *and *PHO84 *were detected for the first time in *G*. *lucidum*. Thirteen (13) potential SSR-motif microsatellite loci were also identified.

**Conclusion:**

The present study demonstrates a successful application of EST analysis in the discovery of transcripts involved in the secondary metabolite biosynthesis and the developmental regulation of *G. lucidum*.

## Background

*Ganoderma lucidum *(Curtis: Fr.) P. Karst, *Lingzhi *in Chinese, which belongs to the *Polyporaceae *family, has been used in China as medicine for centuries to promote health and longevity [1,2]. In other countries, its fruiting body is used to treat a variety of ailments, such as cancers, hypertension, diabetes, and hepatitis, apart from being a dietary supplement [2-4]. *G. lucidum *is an anti-tumour agent that acts via immune modulation or stimulating cytokine production [5-7]. The bioactive constituents of *G. lucidum *include more than 120 different triterpenes and polysaccharides, proteins and other compounds [2,8].

Genes involved in the triterpenoids biosynthesis pathways in *G. lucidum *including squalene synthase (*SQS*), farnesyl-Diphosphate Synthase (*GlFPS*) and HMG-CoA reductase (*Gl -HMGR*) were isolated and characterized [9-11]. Joo *et al*. identified a laccase gene (*GLLac1*) from *G. lucidum *[12]. However, little is known about the molecular biology of its fruiting body and its secondary metabolism. Identification of expressed genes, in particular the transcript profile, of the *G. lucidum *fruiting body would be a key to understanding its molecular biology.

Expressed sequence tag (EST) analysis allows rapid and large-scale identification of uniquely expressed genes [13,14]. The EST analysis was used in transcriptome analysis of *Lentinula edode *[15], *Aspergillus niger *[16], *Ustilago maydis *[17] and *Neurosphora crassa *[18]. Sequencing information from ESTs may help discover genes in the biosynthesis of secondary metabolites [19]. Loo *et al*. identified a gene involved in the ricinoleic acid biosynthetic pathway [20]. Recently, genes encoding enzymes involved in the biosynthesis of ginsenoside, triterpene saponin and diterpenes were identified [21-23]. EST sequencing identified simple sequence repeats (SSRs) for genetic mapping [24].

Using the EST analysis, the present study annotated functional genes involved in the biosynthesis of secondary metabolites and the developmental regulation of the fruiting body of *G. lucidum*. Unique sequences very similar to squalene epoxydase (SE) and farnesyl-diphosphate synthase (FPS) in this EST collection were identified. We also discussed several candidate transcripts possibly associated with the cellular development of *G. lucidum*, such as *hydrophobin*, *MOB2*, *profilin *and *PHO84*. Moreover, identifying SSRs in the EST data is useful in marker-assisted breeding programs.

## Methods

### RNA extraction and cDNA library construction

The fruiting body of *G. lucidum *was obtained from the co-author Jin Lan, who has long been engaged in Ganoderma research in the Institute of Medicinal Plant Development, Chinese Academy of Medical Sciences and Peking Union Medical College, Beijing, China. She authenticated the *G. lucidum *using the morphological identification approach and referred to the Fungi Identification Manual [25]. Fifty (50) days after growing on the basswood medium at 25-30°C in a shade shelter, approximately 0.5 g was harvested and frozen in liquid nitrogen immediately. The mRNA of thefruiting body was isolated and purified directly with a Dynabeads (R) mRNA DIRECT™ kit (Invitrogen, USA) according to the manufacturer's recommendations. The cDNA library was constructed from purified mRNA with a Creator™ SMART™ cDNA Library Construction kit (Clontech, USA). The double-stranded cDNA was directionally ligated into the S*fi *I restriction site of the pDNR-lib vector (Clontech, USA) and electroporated into a DH5α *Escherichia coli *strain (TakaRa, Japan).

### EST sequencing, assembly and annotation

A total of 1,023 randomly selected clones were cultured in liquid LB medium containing 34 mg/l chloramphenicol and incubated overnight at 220 rpm in rotation and 37°C. Plasmid DNA was prepared with an Axyprep-96 plasmid kit (Axygen, USA). The plasmid DNA was submitted for direct sequencing from the 5' end with an M13 forward primer on an ABI 3730 DNA sequencer using BigDye 3.1 sequencing chemistry (Applied Biosystems, USA).

The ABI-formatted chromatogram sequences were processed automatically with a local EST analysis pipeline. The Phred/Phrap program was applied for trace files conversion and for base calling with quality assessment [26,27]. The vector and low-quality regions were removed from the sequence with the Cross Match typically included in the Phred/Phrap program. The short sequences (less than 100 bp) and poly A/T tails were filtered from the EST database. The high quality ESTs were assembled into contigs (clusters of assembled ESTs) and singletons (sequences found only once) by Phrap [28].

The unique sequences were searched against public databases including the SwissProt [29], NCBI non-redundant protein (Nr) [30] and non-redundant nucleotide (Nt) [31] databases using BLAST [32] algorithm, with a *E*-value cut-off at 10^-5^. The functional categories of these unique sequences were classified by a broad category, including metabolism, energy production, cell signalling, cell defence and stress response, cell structure and growth, transcription, protein synthesis, protein degradation, transport and secretion as well as unclassified and unknown function.

### SSR detection

The detection of simple sequence repeats (SSRs) from the total high-quality ESTs of the fruiting body of *G. lucidum *was performed with the Simple Sequence Repeat Identification Tool (SSRIT) [33]. The SSRIT accepts FASTA-formatted sequence files and reports the sequence ID, SSR motif, number of repeats (di- and tri-nucleotide repeat units), repeat length and position of the SSR and the total length of the sequence in which the SSRs were found [34]. The search parameters for the maximum motif-length group were set to hexamer and those for the minimum number of repeats were set to five.

## Result and discussion

### General characteristics of *G. lucidum *fruiting body cDNA library and ESTs

A cDNA library was constructed from the fruiting body of *G. lucidum *for the identification of the transcripts and the expression profiles involved in its cellular development and biosynthesis of secondary metabolites. The cDNA library had a titre of 1.25×10^6 ^colony forming units per millilitre (ml). A total of 1,023 cDNA clones were randomly selected from the library for sequencing, yielding 879 (85.9%) high-quality ESTs after vector screening and short sequence (<100 bp) filtering. These ESTs were assembled into 82 contigs and 518 singletons for a total of 600 unique sequences (Table 1). The average sequence length of these unique sequences was 288 bp, ranging from 0.15 kb to 1.5 kb. Over 63.4% contigs had two sequences, followed by 22.0% having three to four sequences and 14.6% having five to 40 sequences. Approximately 31.75% of the ESTs were redundant. A total of 427 unique sequences (71.1%) displaying no similarities to any sequences in the public databases were probably new transcripts. The redundancy (31.75%) of the ESTs suggests considerable potential for new transcripts in continued sequencing of random colonies from this cDNA library. The sequenced transcripts have been deposited in the GenBank database (GO447131-GO448009).

**Table 1:** Overview of the characteristics of the cDNA library of *G*. *lucidum *fruiting body

Description	Number
Total number of clones sequenced	1,000
Total high quality ESTs	879
Total unique genes	600
Average length per unique sequence (bp)	288
Number of contigs	82
Number of singletons	518
Redundancy (%)	31.75
Number of annotated unique sequences	173
Number of non-annotated unique sequences	427

### Expressed profile of the unique sequences

The expressed profile of the unique sequences identified in the *G*. *lucidum *fruiting body is shown in Table 2. Among 600 unique sequences, 518 (86.3%) unique sequences were sequenced only once; 72 (12%) unique sequences 2-5 times; five (0.8%) unique sequences 6-10 times and five (0.8%) unique sequences 12 times or more. The most abundantly expressed unique sequences in the *G*. *lucidum *fruiting body were coded for the hypothetical proteins (48 ESTs) and the cell wall-associated hydrolase (36 ESTs) (Table 3). Moreover, the unique sequence consisting of five ESTs and with sequence similarity to hydrophobin 2 of *Lentinula edodes *(*Xianggu*) was identified for the first time in *G*. *lucidum *(Table 3). Moreover, the unique sequences matched the elongation factors and the ribosomal proteins were also expressed at high levels (Table 3).

**Table 2:** Occurrence of ESTs in unique sequences

Number of ESTs in a unique sequence	Number of unique sequences
1	518
2	52
3	13
4	5
5	2
6	1
7	1
8	2
10	1
12	1
13	1
22	1
36	1
48	1

**Table 3:** Highly expressed transcripts in *G. lucidum *fruiting body cDNA library

No.of ESTs	BLASTX annotation	E-value
48	Hypothetical protein [*Rattus norvegicus*]	2.00^-16^
36	Cell wall-associated hydrolase [*Capnocytophaga sputigena Capno*]	2.00^-14^
8	Predicted protein [*Coprinopsis cinerea okayama7#130*]	1.00^-11^
8	Protein TAR1 [*Kluyveromyces lactis*]	8.00^-21^
5	Hydrophobin 2 [*Lentinula edodes*]	3.00^-16^
5	Elongation factor 1-alpha [*Schizophyllum commune*]	2.00^-70^
4	Predicted protein [*Laccaria bicolor S238N-H82*]	1.00^-28^
3	Predicted protein [*Laccaria bicolor S238N-H82*]	3.00^-68^
3	Acyl-CoA-binding protein [*Chaetophractus villosus*]	8.00^-21^
3	Elongation factor 2 [*Debaryomyces hansenii*]	8.00^-73^
3	40S ribosomal protein S11 [*Schizosaccharomyces pombe*]	3.00^-57^

### Annotation of expressed sequence tags

The list of the annotated ESTs found in the fruiting body of *G. lucidum *is shown in Additional file 1. Sixty-two (62) ESTs showed sequence similarities to uncharacterized genes encoding hypothetical proteins that were omitted from the list. The unique sequences from this cDNA library were analyzed for similarities by performing BLAST searches against public databases, including SwissProt [29], Nr [30] and Nt [31]. A total of 139 (23.2%) and 67 (11.2%) unique sequences were assigned a putative identity based on significant sequence similarities to at least one sequence in the Nr and Nt databases, respectively. These annotated unique sequences provide an available resource for application and basic microbiology. Furthermore, among the 879 ESTs only three (0.3%) ESTs were identified as homologues of previously reported nucleotides from *G*. *lucidum *in the GenBank database, indicating that the vast majority of the ESTs in our dataset were unique and new. The three unique sequences showed similarities to cytochrome c oxidase subunit 2 (GO447869), glyceraldehyde-3-phosphate dehydrogenase (GO447698) and FPS (GO447502) (Additional file 1).

### Functional distribution of ESTs

**Figure 1:**
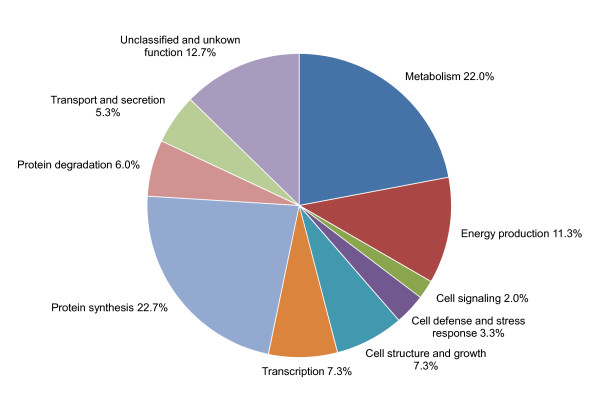
**Distribution of ESTs by broad functional categories**. The functional categories include: metabolism, energy production, cell signaling, cell defence and stress response, cell structure and growth, transcription, protein synthesis, protein degradation, transport and secretion, and unclassified and unknown function.

The functions of the proteins that the identified sequences encoded were classified into categories of metabolism, energy production, cell signalling, cell defence and stress response, cell structure and growth, transcription, protein synthesis, protein degradation, transport and secretion as well as unclassified and unknown functions. The functional distribution of identified sequences from the *G*. *lucidum *fruiting body cDNA library is shown in Figure 1. The unique sequences associated with metabolism (22%), protein synthesis (22.7%), unclassified and unknown function (12.7%) and energy production (11.3%) were strongly represented, whereas those with cell signalling (2.0%) and cell defence and stress response (3.3%) were not. In summary, a total of 173 unique sequences showed similarities to known genes involved in the biosynthesis of secondary metabolites and developmental regulation. While the EST sequencing scale is limited, it provides some information about the expressed transcript profile of the fruiting body of *G. lucidum*.

### SSR detection

SSRs, also known as microsatellites, are useful genetic markers in molecular biology. A total of 13 SSR motifs were identified from the EST sequences of the fruiting body of *G. lucidum *(Table 4). The composition of di- and tri-nucleotide SSRs included AC (two), AT (two), CT (one), GA (one), GT (one), TA (one), TC (one), and TG (two). Only two tri-nucleotide repeats with the composition of CGA and GGT were found in the EST dataset (Table 4). Each SSR-containing unique sequence contains only one SSR motif. The lengths of eight repeats were nine bases and the other five repeats were between 11 and 15 bases. In addition, the tetra-, penta- and hexa-nucleotide repeats were not present in this EST dataset. There was a wide variation in the frequency of SSR motifs among species [35].

**Table 4:** The di- and tri-nucleotide repeats in *G.lucidum *fruiting body ESTs

SSR-containing EST	Motif	Repeat No.	SSR start	SSR end	SSR length	Sequence length
GO447147	AC	5	246	255	9	340
GO447693	AC	8	133	148	15	160
GO447650	AT	5	64	73	9	202
GO447380	AT	5	45	54	9	180
GO447641	CT	5	315	324	9	402
GO447753	GA	7	71	84	13	131
GO447502	GT	6	86	97	11	172
GO447423	TA	5	138	147	9	158
GO447358	TC	5	299	308	9	520
GO447397	TG	5	12	21	9	180
GO447977	TG	5	96	105	9	131
GO447241	CGA	5	88	102	14	293
GO447840	GGT	5	38	52	14	195

### Candidate genes involved in the biosynthesis of triterpenoids

EST analysis is an important tool to identify secondary metabolite genes in the fruiting body of *G. lucidum*. Triterpenoids, the major bioactive compounds in *G. lucidum*, are synthesized from acetyl-CoA in the isoprenoid pathway. While genes involved in the triterpenoid biosynthetic pathway including *SQS*, *GlFPS *and *Gl*-*HMGR *were cloned from and identified in *G. lucidum *[9-11], other genes for the key enzymes in this pathway are to be identified.

According to the studies of the triterpene biosynthesis [10,36], SE and FPS are rate-limiting enzymes in catalyzing triterpenoid biosynthesis in *G. lucidum*. The unique sequence (GO447913) with 71% identity (*E*-value = 1.00^-12^) to SE and the unique sequence (GO447502) with 98% identity to FPS (*E*-value = 4.00^-27^) involved in triterpenoid biosynthesis were presented in our EST data. SE acts as an important regulatory enzyme in the triterpenoid biosynthetic pathway. SE (EC 1.14.99.7), a monooxygenase, converts squalene into 2,3-oxidosqualene [36,37]. The enzyme requires molecular oxygen, flavin adenine dinucleotide (FAD), either NADH or NADPH depending on the organisms [38]. Since the gene encoding SE has not been identified in *G. lucidum*, the information of the unique sequence (GO447913) will help identify and characterize the SE in *G. lucidum*. The EST for FPS (GO447502) shows sequence similarity to the *GlFPS*, suggesting that this EST is the partial sequence of the full-length *GlFPS*.

The genes encoding key enzymes involved in the triterpenoid biosynthesis, such as *SQS*, *Gl*-*HMGR *and others, are not present in this EST dataset, indicates a low abundance of these genes in the fruiting body of *G. lucidum *or incomplete sequencing of the library. The absence of ESTs associated with the polysaccharide biosynthesis, which should be abundant in *G. lucidum*, in this study may be due to the limited sequencing scale.

### Candidate genes involved in regulation of *G. lucidum *development

Several transcripts present in the EST dataset encode the proteins that may be associated with the development processes of the fruiting body of *G. lucidum*. The unique sequence (GO447641) showed sequence similarity to the inorganic phosphate transporter *PHO84 *gene which controls the absorption of phosphate nutrition and regulates the development of *Saccharomyces cerevisiae *[39]. *MOB2 *is a nonessential yeast gene and plays a role in the maintenance of ploidy [40]. The unique sequences homologous to MOB2 (GO447972) and PHO84 (GO447641) may have the same functions as those in yeast. Hydrophobin is expressed specially in filamentous fungi and is important during the morphogenesis of fungi and the fruiting body development of mushrooms [41]. The unique sequence homologous to hydrophobin 2 of *Lentinula edodes *in this cDNA library consisted of five ESTs (GO447695, GO447166, GO447364, GO447414, GO447512), suggesting its abundance in the fruiting body of *G. lucidum*. Suizu *et al*. (2008) reported that the ESTs for hydrophobins were also most frequently identified in the cDNA library of *Lentinula edodes *[15]. Profilin is a universal small eukaryotic protein that binds to monomeric actin (G-actin) and is involved in diverse functions such as maintenance of cell structural integrity, cell mobility and growth factor signal transduction [42]. The sequences (GO447955, GO447282) encoding profilin were present in the *G. lucidum *cDNA library. The important unique sequence encoding an argonaute-like protein (GO447302) may be involved in the RNAi pathway, suggesting a potential gene knock-out by RNA interference in *G. lucidum*. Cloning and characterization of these candidate genes is under way.

### Limitations of the study

The ESTs sequenced in this study from the fruiting body of *G. lucidum *were insufficient to cover all functional genes, although this EST dataset showed some characteristics of gene expression in the fruiting body of *G. lucidum*.

## Conclusion

The present study used EST analysis and identified the transcripts in the biosynthesis of secondary metabolites and the developmental regulation of *G. lucidum*. For example, the candidate transcript encoding SE, the rate-limiting enzyme in the triterpenoid biosynthesis, was identified. Several genes associated with the development processes of *G. lucidum*, such as hydrophobin, MOB2, profilin and PHO84, were also identified.

## Abbreviations

BLAST: Basic Local Alignment Search Tool; bp: base pair; cDNA: complementary DNA; EST: expressed sequence tag; FPS: farnesyl-diphosphate synthase; HMGR: HMG-CoA reductase; NCBI: National Center for Biotechnology Information; Nr: NCBI non-redundant protein; Nt: NCBI non-redundant nucleotide; SE: squalene epoxidase; SQS: squalene synthase; SSRs: simple sequence repeats

## Competing interests

The authors declare that they have no competing interests.

## Authors' contributions

HML analyzed the data and drafted the manuscript. CS and YL participated in the data analysis. JYS participated in the study design. JL and XWL collected tissue samples. SLC evaluated the results and revised the manuscript. All authors read and approved the final version of the manuscript.

## Supplementary Material

Additional file 1 **Putative functions of partial *G. lucidum *fruiting body ESTs**. This table summarizes putative functions of partial ESTs of *G. lucidum *fruiting body. Click here for file
