# Supplementary material for: Generation and analysis of expressed sequence tags from a cDNA library of the fruiting body of Ganoderma lucidum
Source: Chin Med. 2010 Mar 16;5:9. doi: 10.1186/1749-8546-5-9 (PMC2848221; doi:10.1186/1749-8546-5-9)
Supplement: Additional file 1 — Putative functions of partial G. lucidum fruiting body ESTs. This table summarizes putative functions of partial ESTs of G. lucidum fruiting body. [file 1749-8546-5-9-S1.DOC]

Putative functions of partial *G*.*lucidum* fruiting body ESTs

| **Putative functions^a^** | **GenBank accession No.^b^** |
| --- | --- |
| **Metabolism** |  |
| 5-hydroxyisourate hydrolase | GO447937 |
| Acyl-CoA-binding protein | GO447449,GO447328,GO447690 |
| Alpha-galactosidase | GO447834,GO447552 |
| Aryl-alcohol dehydrogenases | GO447181 |
| FKBP-type peptidyl-prolyl cis-trans isomerase | GO448005 |
| Flavin-binding monooxygenase | GO447408 |
| Farnesyl-diphosphate synthase | GO447502 |
| GCN5-related N-acetyltransferase | GO447756 |
| Glyceraldehyde-3-phosphate dehydrogenase | GO447698 |
| Glycoside hydrolase family 5 protein | GO447979 |
| Glycosyltransferase family 2 protein | GO447209 |
| Hexokinase | GO447901 |
| Malate dehydrogenase | GO447797 |
| NADPH-dependent D-xylose reductase | GO447793 |
| Phosphatidylglycerol/phosphatidylinositol transfer protein | GO447448,GO447175 |
| Phosphoglycerate kinase | GO447982,GO447839 |
| Predicted ornithine decarboxylase antizyme | GO447804,GO447974,GO447791 |
| Predicted thioesterase superfamily | GO447388 |
| Pyruvate carboxylase | GO447424 |
| Pyruvate kinase | GO447200,GO447813 |
| SMP3 mannosyltransferase | GO447941 |
| Squalene epoxidase | GO447913 |
| Sulfate permease 2 | GO447487 |
| Zinc-binding dehydrogenase | GO447905,GO447853 |
| **Energy production** |  |
| Aminophospholipid-transporting P-type ATPase | GO447443 |
| ATP synthase subunit gamma | GO447667 |
| Cytochrome b2 | GO447164 |
| Cytochrome b5 | GO447223 |
| Cytochrome c oxidase-assembly factor COX23 | GO447559 |
| Cytochrome c oxidase subunit 2 | GO447869 |
| Hypothetical protein Csac_2309 | GO447521,GO447795 |
| Mitochondrial import inner membrane translocase | GO447347 |
| NADH-cytochrome b5 reductase | GO447904 |
| NADH dehydrogenase [ubiquinone] iron-sulfur protein | GO447451 |
| NADH-ubiquinone oxidoreductase 12 kDa subunit | GO447615 |
| NADH-ubiquinone oxidoreductase 21 kDa subunit | GO447585 |
| NADH-ubiquinone oxidoreductase 21 kDa subunit | GO447598,GO447242 |
| NifU-like protein C1709.19c | GO447384 |
| Predicted protein | GO447975 |
| Presequence translocated-associated motor | GO447440 |
| **Signaling** |  |
| Ras2 | GO447219 |
| Serine-threonine kinase receptor-associated protein | GO447947,GO447855 |
| **Cell defence, stress** |  |
| Cytochrome P450 | GO447162 |
| Cytochrome P450 | GO447247 |
| Glutathione transferase | GO447530,GO447631 |
| Predicted cytochrome P450 | GO447510 |
| **Cytoskeleton** |  |
| Profilin | GO447955,GO447282 |
| Tubulin beta chain | GO447554,GO447971 |
| **Cell growth** |  |
| Hydrophobin 2 | GO447695,GO447166,GO447364,  GO447414,GO447512 |
| Maintenance of ploidy protein mob2 | GO447972 |
| Meiotic recombination-related protein | GO447658 |
| **DNA, RNA, chromatin** |  |
| Chromatin modification-related protein EAF3 | GO447240 |
| DNA-directed RNA polymerases I, II, and III subunit RPABC5 | GO447617 |
| DNA polymerase delta catalytic subunit | GO447227 |
| Histone H2B | GO447990,GO447664 |
| Histone H4 | GO447909 |
| Nuclear transport factor 2 | GO447229 |
| Predicted DNA polymerase epsilon subunit B | GO447812 |
| rRNA intron-encoded homing endonuclease | GO447946,GO447474 |
| Small nuclear ribonucleoprotein Sm D2 | GO447369,GO447991 |
| **Transcription/Transcript processing** |  |
| Argonaute-like protein At2g27880 | GO447302 |
| Predicted high mobility group box | GO447518 |
| Pre-mRNA-splicing factor 18 | GO447934 |
| Protein TAR1 | GO447298,GO447307,GO447453,  GO447824,GO447257,GO447818,  GO447140,GO447191 |
| **Translation** |  |
| 40S ribosomal protein S6-B | GO447945 |
| 40S ribosomal protein S11 | GO447825,GO447860,GO447655 |
| 40S ribosomal protein S12 | GO447332 |
| 40S ribosomal protein S18 | GO447517 |
| 40S ribosomal protein S23 - | GO447632 |
| 60S ribosomal protein L32 | GO447691 |
| Hypothetical protein CC1G_00382 | GO447464 |
| Elongation factor 1-alpha | GO447236,GO447500,GO447850,  GO447709, GO447923 |
| Elongation factor 2 | GO447666,GO447189,GO447800 |
| Elongation factor 3 | GO447957 |
| Eukaryotic translation initiation factor 3 39 kDa subunit | GO447406 |
| Eukaryotic translation initiation factor 6 | GO447852 |
| Signal peptidase complex catalytic subunit SEC11C | GO447390 |
| Signal recognition particle receptor subunit alpha | GO447730 |
| **Protein degradation** |  |
| 20S proteasome subunit | GO447842 |
| Peptidase | GO447435,GO447472 |
| Proteasome activator complex subunit 3 | GO447155 |
| Small ubiquitin-related modifier precursor | GO447401 |
| T-complex protein 1 subunit beta | GO447890,GO447858 |
| Ubiquitin | GO447587 |
| Ubiquitin-protein ligase | GO447353 |
| **Transport, secretion** |  |
| Acyl carrier protein | GO447914,GO447966 |
| Carnitine/acyl carnitine carrier | GO447171,GO447457 |
| Hypothetical protein CC1G_03241 | GO447205 |
| Inorganic phosphate transporter PHO84 | GO447641 |
| OPT oligopeptide transporter | GO447778 |
| Protein SSO2 | GO447938 |
| **Unclassified** |  |
| Anamorsin | GO447473 |
| Cat eye syndrome critical region protein 1 | GO447745 |
| DNA-binding TFAR19-related protein | GO447466 |
| Endosomal protein P24B precursor | GO447790 |
| FK506-binding protein 1 | GO448005 |
| NSFL1 cofactor p47 | GO447657 |
| RWD domain-containing protein 1 | GO447647 |
| Small secreted protein | GO447592 |
| **Unkown function** |  |
| Predicted protein | GO447393,GO447349 |
| Predicted protein | GO447245,GO447144,GO447816,  GO447910,GO447844,GO447725,  GO447222,GO447551 |
| Predicted protein | GO447700 |

Note: ^a^ The putative function annotated by BLASTX analysis against the public databases are classified by a broad category; ^b^ The accession number of GenBank.
